# Comparison of alternate part-time patching and pencil push-up training for patients with intermittent exotropia

**DOI:** 10.1186/s12886-022-02705-3

**Published:** 2022-11-29

**Authors:** Desheng Song, Ling Yin, Danni Chen, Jing Qian, Zhijun Chen

**Affiliations:** 1 Department of Ophthalmology, Children’s Hospital of Nanjing Medical University, Nanjing, 210008 China; 2 Department of Ophthalmology, The First Hospital of Changsha, Changsha, 410005 China

**Keywords:** Intermittent exotropia, Alternate part-time patching, Pencil push-up training

## Abstract

**Background:**

To compare the effect of alternate part-time patching and pencil push-up training on control ability in patients with intermittent exotropia.

**Methods:**

Patients (3–7 years old) with previously untreated intermittent exotropia were randomly assigned to receive alternate part-time patching, pencil push-up training, or observation. Control ability was assessed using the Office Control Score. Stereoacuity at 40 cm was evaluated with Titmus. Results were compared after a 12-week follow-up.

**Results:**

Ninety-two patients (28 in patching, 30 in pencil push-ups, and 34 in observation group) completed 12-week follow-up assessments. Based on 6-point scale, the mean deviation control was significantly better in patching and pencil push-up group after 12 weeks at distance (*P* = 0.002 and 0.026, respectively). Furthermore, there were greater control changes in patching and pencil push-up groups in comparison with observation group from baseline to 12 weeks (*P*<0.001; *P* = 0.003, respectively). After 12 weeks of treatment, stereoacuity and stereoacuity changes were not significantly different between either the intervention group or control group (*P* = 0.140 and 0.393, respectively).

**Conclusions:**

Based on the common office control scale, alternate part-time patching and pencil push-up training were effective treatment strategies for intermittent exotropia.

Intermittent exotropia (IXT) is the most common type of strabismus and it is estimated that approximately 3% of Chinese teenagers suffer from this condition [1]. Intermittent exotropia is generally divided into four types based on distance/near differences: basic, true divergence excess, pseudo-divergence excess, and convergence insufficiency. A range of nonsurgical and surgical treatment options can be employed in the management of intermittent exotropia, and includes correction of refractive error, patching, orthoptic therapy, prism therapy, overminus lens therapy and surgery [2]. Current evidence shows that nonsurgical treatment improves fusional amplitudes, but does not reduce the angle of deviation. Surgical intervention is meant to reduce the angle of deviation and may therefore improve the control of the deviation [3–5]. Control ability has important clinical application value to assess the impact of nonsurgical treatment on intermittent exotropia [6, 7]. Compared to other nonsurgical treatment methods, alternate patching and pencil push-up training are easy-to-operate and inexpensive therapeutic modalities. Pencil push-ups are the most commonly prescribed treatment for convergence insufficiency intermittent exotropia by both optometrists and ophthalmologists [8]. In our previous studies, pencil push-ups were found to be effective in children with basic and divergence excess intermittent exotropia as well [9]. This study aims to assess the effectiveness of alternate part-time patching and pencil push-up training on the control ability of patients with intermittent exotropia.

## Methods

The study was approved by Nanjing Children’s Hospital Review Board, and the parent or guardian of each subject signed the informed consent prior to treatment.

### Eligibility criteria

In this study, the ages of subjects ranged from 3 to 7. The spherical equivalent refraction was between − 6.00 diopters(D) and + 1.00D. And patients diagnosed as intermittent exotropia met the following criteria: (1) intermittent exotropia or constant exotropia at distance (the distance control assessment at baseline was performed three times and the average value ≥2 points); (2) intermittent exotropia or exophoria at near (the near control assessment at baseline was performed three times, at least one time ≤ 4 points); (3) ocular deviation measured using prism and alternate cover test (PACT) was more than 15PD at distance or near, but at least 10PD at distance. Children who received nonsurgical treatment within 6 months were excluded.

### Enrollment tests

Patients underwent a complete ophthalmic evaluation before enrollment. Exodeviation control ability was measured at distance (6 m) and near (33 cm) using a 6-point office control score (Table 1) [10, 11] which ranked from 0 (best control) ~ 5 (worst control). Control score was measured three times within 1 day because of its large variability. Stereoacuity at 40 cm was measured using Titmus stereo test.

**Table 1:** Exotropia control assessment procedure

Control	Score
constant exotropia	5
exotropia > 50% of the 30-seconds period before dissociation	4
exotropia<50% of the 30-seconds period before dissociation	3
no exotropia unless dissociated, recovers in > 5 seconds	2
no exotropia unless dissociated, recovers in 1–5 seconds	1
no exotropia unless dissociated, recovers in<1 second (phoria)	0

### Treatment regimens

Participants were randomly assigned to one of the three groups: patching, pencil push-ups, and observation. All exams were completed by a masked examiner.

Part-time patching: For intermittent exotropia with equal dominance, eyes were covered alternately for 12 weeks, and 2 hours every day. For intermittent exotropia with dominant eye, the dominant eye was covered for 4 days and non-dominant eye for 3 days per week, and 2 hours every day. In the case of changing dominancy during treatment, the patching regimen was altered accordingly.

Pencil push-up training: patients were instructed to hold a pencil at arm’s length distance along the midline, and an index card on the wall behind the pencil was used to control suppression by using physiological diplopia. Patients were instructed to look at the tip of the sharpened pencil and to try and keep the pencil point single while moving it toward their nose. When they perceived double image of the target even with maximum effort, the pencil was moved back slowly until they regained fusion. If suppression occurred and one of the physiologic diplopia images disappeared, the subjects were instructed to blink or shake the pencil as an antisuppression technique. If patients were able to regain a single vision, they were asked to continue moving the pencil closer, up to 5 cm from their nose. Patients were instructed to do this exercise for 3 sets of 20 pencil push-ups daily, 5 days per week [12].

Observation: patients in the observation group did not receive any intervention except refractive correction.

### Follow-up visits

Patients were followed up for 12 weeks. The compliance for training and patching was assessed after discussing with the parents and by reviewing study calendars on which parents recorded the number of hours the child patched and the number of training sessions each day. Compliance was judged to be excellent (the percentage of the number of training sessions over the total number or covering time more than 75%), good (more than 50% to less than or equal 75%), fair (more than 25% to less than or equal 50%), or poor (less than or equal 25%). Control scores and stereopsis at 40 cm were then evaluated. Stereopsis became a continuous variable by converting the seconds of arc scores to log arc/sec values, for example, 40 (1.60), 50 (1.70), 60 (1.78), 80 (1.90), 100 (2.00), and 200 (2.30). Stereopsis threshold doubled (e.g. 100 to 200 arc/sec) with 0.3 change in log transformed value.

During the 12-week visits, patching compliance was observed to be excellent in 15 patients (54%), good in eight patients (29%), fair in four patients (14%), and poor in one patient (4%). Pencil push-up training compliance was observed to be excellent in 17 patients (57%), good in seven patients (23%), fair in three patients (10%) and poor in two patients (7%), and unknown in one patient (3%).

Throughout the follow-up period, unilateral patching was prescribed in eight participants (29%), alternate patching in 18 participants (64%), and both alternate and unilateral patching at different follow-up periods in two participants (7%).

### Statistical analysis

Data was normally distributed and was analyzed using SPSS 19.0. Paired t-test was used to compare deviation control before and after the intervention. Chi-square test or Fisher’s exact test was used to compare counting data. One-way ANOVA followed by post hoc tests was used to compare measurement data. *P*-value less than 0.05 was considered statistically significant.

## Results

A total of 108 patients were eligible for the trial. Among them, 92 patients (28 in patching, 30 in pencil push-ups, and 34 in observation group) completed the follow-up. Poor adherence to patching or pencil push-up regimen and loss of follow-up were the main reasons for dropout. There were 45 male patients and 57 female patients and the average age was 5.23 ± 1.66 years (3–7 years). Thirty-six participants had significant fixation dominancy. Being informed by their parents, 27 patients had photophobia. The best corrected visual acuity (BCVA) for all cases was 10/10, and corrective lenses were worn by 16 subjects. Basic information on patient data is shown in Tables 2 and 3. No significant difference in the baseline values was observed among the three groups.

**Table 2:** Demographic characteristics of patients

Variables	Patch(*n* = 28)	Pencil push-up(*n* = 30)	Observation(*n* = 34)	*P*-value
Age (mean ± SD)	5.45 ± 1.45	5.24 ± 1.42	5.66 ± 1.37	0.482^a^
Sex (male:female)	16:12	14:16	15:19	0.767^b^
Spherical equivalent (mean ± SD)	1.14 ± 0.55	1.08 ± 0.98	1.12 ± 0.87	0.552 ^a^
Significant dominancy (%)	10 (36)	8 (27)	12 (35)	0.407 ^b^
photophobia (%)	8 (29)	10 (33)	9 (26)	0.377 ^b^

^a^Based on *t* test

^b^Based on the chi-square test

**Table 3:** Baseline exotropia control by treatment group

	Distance control			Near control		
	G1(28)	G2(30)	G3(34)	G1(28)	G2(30)	G3(34)
	N(%)	N(%)	N(%)	N(%)	N(%)	N(%)
control						
0	0	0	0	5 (18)	7 (23)	11 (32)
1	0	0	0	10 (36)	12 (40)	9 (26)
2	16 (57)	12 (40)	16 (47)	9 (32)	6 (20)	6 (18)
3	4 (14)	7 (23)	12 (35)	1 (4)	2 (7)	4 (12)
4	5 (18)	7 (23)	3 (9)	2 (7)	1 (3)	2 (6)
5	3 (11)	4 (13)	3 (9)	1 (4)	2 (7)	2 (6)
Mean (SD)	2.8 (1.1)	3.1 (1.1)	2.8 (0.9)	1.6 (0.2)	1.5 (0.3)	1.5 (0.5)

G1: alternate patching group; G2: pencil push-ups group; G3: observation group

### Office control score

The distance control improved significantly in the intervention groups. It decreased from 2.8 ± 1.1 points to 1.6 ± 1.0 points (*P*<0.001) in the alternate patching group and from 3.1 ± 1.1 to 2.0 ± 1.5 (*P* = 0.002) in the pencil push-ups group. There was no significant improvement in the observation group (2.8 ± 0.9 points VS. 2.6 ± 1.4 points; *P* = 0.486).

A comparison of the mean values at the end of treatment demonstrated a significant difference among the three groups (*P* = 0.013). Post hoc testing revealed that the mean distance control for the alternate patching group and pencil push-ups group was significantly greater than the mean of observation group (*P* = 0.002; *p* = 0.026). There was no significant difference when comparing the alternate patching group with pencil push-ups group (*P* = 0.134) (Table 4).

**Table 4:** Exotropia control at 12-week outcome (Average of 3 Measurements)

	Distance control			Near control		
	G1(28)	G2(30)	G3(34)	G1(28)	G2(30)	G3(34)
	N(%)	N(%)	N(%)	N(%)	N(%)	N(%)
control						
0	1 (4)	4 (13)	1 (3)	8 (43)	8 (50)	10 (24)
1	15 (54)	11 (37)	7 (21)	11 (29)	14 (27)	8 (21)
2	7 (25)	6 (20)	11 (32)	8 (21)	4 (13)	7 (21)
3	4 (14)	2 (7)	4 (12)	1 (4)	2 (3)	5 (15)
4	0	5 (17)	7 (21)	0	1 (3)	2 (12)
5	1 (4)	2 (7)	4 (12)	0	1 (3)	2 (9)
Mean (SD)	1.6 (1.0)	2.0 (1.0)	2.6 (1.1)	1.0 (0.2)	1.2 (0.2)	1.6 (0.3)
Change from baseline to 12 weeks (points)						
Mean (SD)	−1.2 (0.9)	−1.1 (1.2)	−0.2 (1.1)	−0.6 (0.2)	−0.3 (0.1)	0.1 (0.2)
Improved 1 point	17 (61%)	20 (67%)	12 (35%)	4 (25%)	4 (27%)	2 (12%)

G1: alternate pacthing group; G2: pencil push-ups group; G3: observation group

Furthermore, the means of control changes in the alternate patching group and pencil push-ups group were larger than that of the observation group (*p*<0.001; *P* = 0.003). However, no significant difference was observed between the alternate patching group and pencil push-ups group (*p* = 0.720) (Table 4).

After 12 weeks, distance control improved one point from baseline in 17 participants (61%) in the alternate patching group, 20 participants (67%) in the pencil push-ups group, and 12 participants (35%) in the observation group (*P* = 0.027). Alternate patching group and pencil push-up group exhibited a better control improvement than observation group (*p* = 0.010; *P* = 0.012). No notable difference was observed between the two treatment groups (*p* = 0.637) (Table 3).

The distance control had more improvement in participants with poorer distance control before treatment. There were suggestions that the posterior treatment difference magnitude (favoring alternate patching and pencil push-ups treatment) was greater in subjects with poorer distance control at baseline (Tables 5 and 6).

**Table 5:** Change in control from baseline to 12 weeks according to baseline distance control

Baseline Distance Control Score (Points)	Change in Control from Baseline to 12 Weeks (Points)					
	G1(28)		G2(30)		G3(34)	
	N	Mean	N	Mean	N	Mean
2- < 3	16	−0.5	12	−0.6	16	0.1
3- < 4	4	−0.8	7	−0.9	12	−0.4
4–5	8	−2.4	11	−2.3	6	−0.9

G1: alternate patching group; G2: pencil push-ups group; G3: observation group

N: The total number of subjects; Mean: the means of distance control score changes

**Table 6:** Treatment response at 12 Weeks

Baseline Distance Control Score (Points)	Treatment Response at 12 Weeks (Control Improved ⩾1 Point)					
	G1(28)		G2(30)		G3(34)	
	N	n(%)	N	n(%)	N	n(%)
2- < 3	16	8 (50)	12	6 (50)	16	4 (25)
3- < 4	4	2 (50)	7	5 (71)	12	6 (50)
4–5	8	7 (87)	11	9 (82)	6	2 (33)

G1: alternate patching group; G2: pencil push-ups group; G3: observation group

N: The total number of subjects; n: the number of patients whose distance control score increased by one point

However, after 12 weeks, there was no difference among the three groups in respect of the control at near (Table 4).

### Stereopsis

Eighty-eight patients cooperated for stereo acuity examination (27 in patching, 29 in pencil push-ups, and 32 in observation group). Means of log arc/sec at baseline were 1.85 ± 0.32 in the alternate patching group, 1.76 ± 0.28 in the pencil push-ups group and 1.88 ± 0.34 in the observation group. After the 12-week treatment, they were 1.72 ± 0.12, 1.69 ± 0.09 and 1.75 ± 0.14, respectively. There was no significant difference among the three groups after treatment (*P* = 0.140). Also, no significant difference was found among the three groups in the difference values of stereoacuity before and after training (*P* = 0.393).

## Discussion

There was a significant improvement in the distance control score after 12 weeks of patching and pencil push-up training compared to observation in children ages 5 to 7 years old. Rather than using observation as a sole treatment modality, this study suggests that non-surgical treatment like alternate patching or pencil push-ups might be beneficial to young patients with intermittent exotropia.

A randomized clinical trial conducted by Mohammad Reza Akbari showed that patching caused more improvement in deviation control from baseline to 3 months and baseline to 6 months at both near and distance [13]. Another application of part-time patching in the treatment of intermittent exotropia was explored by the Pediatric Eye Study Group (PEDIG), which examined the effectiveness of part-time patching in preventing deterioration in better-controlled patients but not improving control in poorly controlled patients. PEDIG found that deterioration beyond 6 months is rare in untreated children with intermittent exotropia between 12 and 35 months or 3 to 10 years old, with or without patching treatment [14, 15]. Pencil push-up training is easy to learn and perform, which may have a considerable effect when combined with intermittent exotropia surgery [8]. The present study found that pencil push-up training and part-time patching had similar therapeutic efficacy in improving distance deviation control of 3–7-year-old children with intermittent exotropia in comparison with observation.

In addition to our primary outcome, we also evaluated the near stereoacuity at 12-week treatment. Aligned with findings in previous research [13–15], no substantial improvement was found in sensory fusion after patching or pencil push-ups. The definition of deterioration of intermittent exotropia based on a decrease in near stereoacuity is unclear. Holmes et al. reported that 6 of 95 untreated children (7%) showed a 2-octave reduction in a single near stereoacuity measurement, and 4 of these children showed a regression to baseline stereoacuity levels at subsequent follow-up, emphasizing the need for confirmatory retesting at the same or subsequent visits [16]. Since the stereoacuity test must be retaken on the same day in our current trial, some patients who have been classified as deteriorating may have tested poorly because they were uneasy or uncooperative that day or because of the intrinsic variability of intermittent exotropia [17]. Nonetheless, due to the small decrease in stereoacuity in the two treatment groups, any overestimated deterioration of stereoacuity as a result of not requiring a retest on a subsequent day would be minimal. In addition, any small overestimation of the deterioration in stereoacuity is unlikely to have affected the comparison of the treatment groups, as the expected overestimation of deterioration is the same for both groups, given that the treatment groups did not differ in terms of change in stereoacuity.

Secondary analyses were performed to assess the effectiveness of patching and pencil push-ups based on the distance control. It is noteworthy that the improvement of distance control was more evident for children with lower baseline distance control than for those with better distance control. The greater response in children with poorer baseline control may be partly due to a return to the average and significant room for improvement. However, the observation group did not exhibit the same level of response, indicating that the larger impact of patching and pencil push-ups on children with poorer baseline control may exist. However, due to the small sample size of the subgroup, this conclusion needs to be interpreted with caution.

Another issue of concern is treatment adherence. In this study, 23 of the 28 (83%) patients in the patching group were judged to have good or excellent adherence, compared with 80% (24/30) in the pencil push-up training group. A slightly lower percentage of patients demonstrated more than 50% good treatment adherence during the short-term follow-up period. It should be noted that there were no differences in the therapists’ assessment of patient adherence between the 2 treatment groups. Not all patients in previous studies showed excellent or good compliance. In a randomized clinical trial on 358 children aged 3 to 10 years with untreated intermittent exotropia, compliance with patching was judged to be excellent or good in 141 participants (88%) at the 6-month outcome visit [15]. Merna found that 27% of patients in the part-time patching therapy group were considered to have good adherence [18]. Scheiman reported that 61.5% of patients in the pencil push-up group performed their home therapy 75% of the time at 8 weeks [19]. In another study, therapists estimated that 73% of the patients performed pencil push-up training 75% of the time at 12 weeks [18]. Considering that children’s low adherence to patching or pencil push-ups may affect treatment outcomes, this issue requires necessary action. Behavior change communication (BCC) aimed at education and behavior change of parents is advocated, and more attention should be paid to children’s psychological status during the treatment.

The primary drawback of this study was the brief follow-up period. Examining the therapeutic importance of patching and pencil push-ups in the treatment of intermittent exotropia is warranted by studies with long-term follow-up. Furthermore, monocular cues are present with the largest disparity levels of the contour-based Titmus tests, which could overestimate steoreopsis in patients. Future work using stereopsis tests like Random Dot or Global stereopsis measures will be more sensitive to bifoveal fixation. Lastly, pencil push-up is the most common treatment for convergence insufficiency intermittent exotropia [8]. Our study included multiple types of intermittent exotropia, which may have resulted in an underestimation of the therapeutic effect of pencil push-ups.

## Data Availability

The datasets generated and analysed during the study are not publicly available but are available from the corresponding author on reasonable request.

## Abbreviations

IXT: Intermittent exotropia
PD: Prism diopter
NCS: Newcastle Control Score
PEDIG: The Pediatric Eye Researchers Group
